# Discovery and functional implications of a miR-29b-1/miR-29a cluster polymorphism in acute myeloid leukemia

**DOI:** 10.18632/oncotarget.23150

**Published:** 2017-12-12

**Authors:** Apollinaire Ngankeu, Parvathi Ranganathan, Violaine Havelange, Deedra Nicolet, Stefano Volinia, Bayard L. Powell, Jonathan E. Kolitz, Geoffrey L. Uy, Richard M. Stone, Steven M. Kornblau, Michael Andreeff, Carlo M. Croce, Clara D. Bloomfield, Ramiro Garzon

**Affiliations:** ^1^ Division of Hematology, Department of Internal Medicine, The Ohio State University, Columbus, OH, USA; ^2^ Department of Molecular Virology, Immunology and Medical Genetics at The Comprehensive Cancer Center, The Ohio State University, Columbus, OH, USA; ^3^ Hematological Department, Cliniques Universitaires Saint-Luc, Brussels, Belgium; ^4^ Comprehensive Cancer Center, The Ohio State University, Columbus, OH, USA; ^5^ Department of Morphology, Surgery and Experimental Medicine, University of Ferrara, Ferrara, Italy; ^6^ The Comprehensive Cancer Center of Wake Forest University, Winston-Salem, NC, USA; ^7^ North Shore Cancer Institute, Lake Success, NY, USA; ^8^ Siteman Cancer Center, Washington University School of Medicine, St. Louis, MO, USA; ^9^ Dana-Farber Cancer Institute, Harvard University, Boston, MA, USA; ^10^ Department of Leukemia, MD Anderson Cancer Center, Texas State University, Houston, TX, USA; ^11^ Alliance for Clinical Trials in Oncology Statistics and Data Center, Mayo Clinic, Rochester, MN, USA

**Keywords:** polymorphism, miR-29b-1/miR-29a cluster, AML

## Abstract

We previously reported that microRNA (miR)-29b is down-regulated and has a tumor suppressor role in acute myeloid leukemia (AML). However, little is known about the mechanisms responsible for miR-29b expression downregulation in AML. In this work we screened for mutations that could affect miR-29b expression. Using Sanger sequencing, we identified a germline thymidine (T) base deletion within the miR-29b-1/miR-29a cluster precursor in 16% of AML patients. Remarkably we found a significant enrichment for the presence of the miR-29 polymorphism in core binding factor (CBF) newly diagnosed AML patients (*n* = 61/303; 20%) with respect to age, sex and race matched controls (*n* = 43/402:11%, *P* < 0.01). Mechanistically, this polymorphism affects the expression ratio of mature miR-29b and miR-29a by dampening the processing of miR-29a. RNA immunoprecipitation assays showed reduced DROSHA binding capacity to the polymorphism with respect to the controls. Finally, we showed that this polymorphism negatively impacts the ability of miR-29b-1/miR-29a cluster to target *MCL-1* and *CDK6*, both known miR-29 targets.

## INTRODUCTION

Acute myeloid leukemia (AML) is a highly heterogeneous malignant disease of the hematopoietic system characterized by a clonal accumulation of immature myeloid precursor cells in the bone marrow (BM) and peripheral blood (PB) [1]. Structural genetic alterations, including gene deletion, inversions and translocations have been found in about 55 to 60% of AML patients [1, 2]. These alterations, for the most part, represent the initial and contributing events leading to malignant transformation [1, 2]. Patients with cytogenetically normal AML (CN-AML) comprise from 30 to 50% of newly diagnosed AML cases and exhibit striking differences at the molecular level resulting in a substantial heterogeneity in terms of clinical outcome [1–5].

Over the past decade, a novel class of small non-coding RNAs, named microRNAs (miRNAs) have been shown to regulate gene expression by binding to target mRNAs of protein-coding genes and inhibiting protein translation and/or causing RNA degradation [6]. Deregulation of miRNAs, and in turn their target genes, has been found to contribute to malignant transformation in several human solid tumors and hematologic malignancies by interfering with the expression of proteins involved in critical cell processes such as development, differentiation, proliferation and apoptosis [6, 7]. We and others have shown unique miRNA expression signatures that are associated with genetic and molecular subsets of AML [8–12]. Among the miRNAs whose expression is deregulated in AML, we have focused on miR-29 family members for several reasons. First, the miR-29 family is composed of 3 isoforms arranged in 2 clusters: miR-29b-1/miR-29a located on chromosome 7q32 and miR-29b-2/miR-29c located on chromosome 1q23. Interestingly, chromosome 7q32 is a region frequently deleted in myelodysplastic syndromes (MDSs) and therapy-related AML [13]. Second, miR-29 family members have been shown to be down-regulated in several cancers including high-risk chronic lymphocytic leukemia (CLL) [14], lung cancer [15], invasive breast cancer [16], cholangiocarcinoma [17] and rhabdomyosarcoma [18]. Third, we have previously shown that miR-29b expression is down-regulated in CN-AML with wild type nucleophosmin (*NPM1*) [19], *c-KIT* mutated core binding factor (CBF) [20], monosomy 7 or del7q [21] and t(11q23) [8] patients. Last, restoration of miR-29b expression in AML cell lines and primary AML blasts induces apoptosis and dramatically reduces tumorigenicity in a xenograft leukemia model [21]. We have also shown that miR-29b is central to regulation of both DNA methylation and tyrosine kinase receptor activities in leukemia cells [20, 22]. Altogether, these studies support a tumor suppressor function for miR-29b in AML.

However, with the exception of AML cases where chromosome 7 is deleted [21], little is known about how miR-29b expression is down-regulated in AML. Since miRNAs expression could be lost due to mutations [14], in this work we screened AML patient samples for mutations that could affect miR-29b expression and function.

## RESULTS

### A thymidine (T) base deletion polymorphism within the miR-29b-1/miR-29a cluster precursor is enriched in core binding factor AML

**Figure 1:**
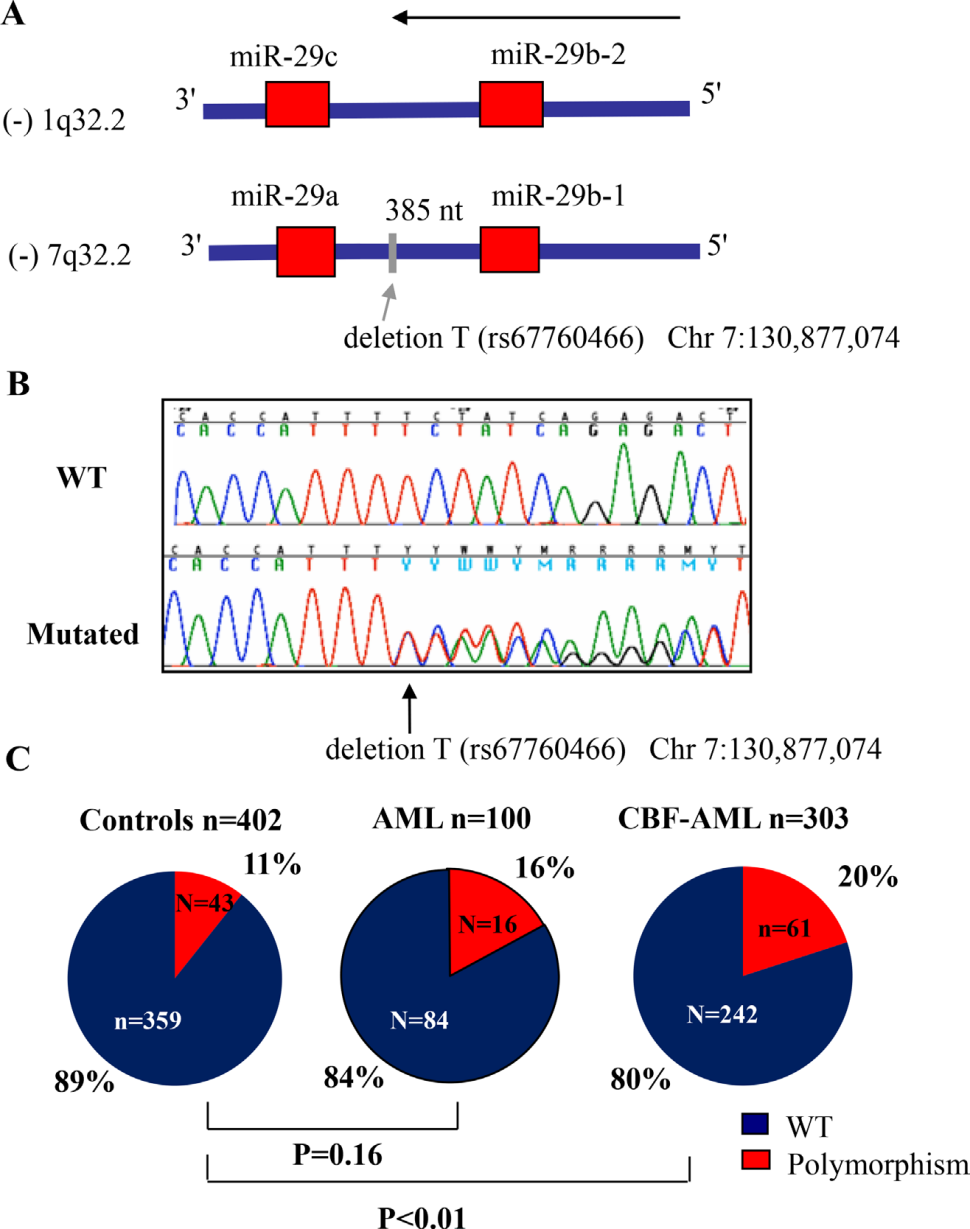
Frequency of thymidine (T) base deletion (rs67760466) in acute myeloid leukemia (AML) (**A**) Diagram showing both miR-29 clusters and the location of the T base deletion. (**B**) Chromatograms showing a wild type (WT) and a polymorphism case. (**C**) Frequency of the polymorphism (rs67760466) in AML patients and in controls. *P* values were obtained using the Fisher's exact test. Patient characteristics are detailed in Tables 1 and 2.

To screen for mutations that may affect miR-29b expression, we sequenced the whole miR-29b-1/miR-29a cluster genomic DNA region located in chromosome 7q32 (including 200 base pairs (bp) at the 5′ of the miR-29b-1 and at the 3′ of the miR-29a precursor, Figure 1A) in a representative cohort of 100 primary AML samples (MD Anderson cohort, See Table 1 for patient characteristics). We focus on this cluster because it is located in a fragile genomic area that is commonly deleted and mutated in hematological malignancies, including AML [13]. Using Sanger sequencing we identified a (T) base deletion within the miR-29b-1/miR-29a cluster precursor miRNAs (at –385 bp from the 3′ position of the miR-29b precursor and –264 bp from the 5′ position of miR-29a in chromosome 7q32) in 16/100 (16%) patients (Chr7:130,877,074delT) (Figure 1B). This deletion correspond to an A base deletion described in the positive (+) strand at the same position (rs67760566) in the dbSNP database; http://www.ncbi.nlm.nih.gov/SNP/. The T base deletion was observed in 3/10 inv(16) and 7/62 CN-AML patients, while the other 6 cases were distributed among complex karyotype (CK) (2/11), isolated –7 (1/5), 11q23 (1/4) and other cytogenetics (2/5). For the most part, the T base deletion was heterozygous (*n* = 14), while in the cases of isolated monosomy 7 and in –7 associated with CK, the remaining alleles have the T base deletion. To investigate whether this genomic finding was somatic or germline, we sequenced the same genomic region but using buccal mucosa DNA from two patients who were identified as positive for the T base deletion. We confirmed that the same heterozygous T base deletion was observed in these two cases, supporting a germline origin (data not shown). Next, to investigate the frequency of this abnormality in the healthy population, we sequenced only the genomic area where this T base deletion was observed (about 200 bp) using genomic DNA obtained from PB mononuclear cells of 402 healthy individuals of similar sex and race to the MD Anderson leukemic cohort, except for White-Hispanics that were more frequent in the MD Anderson cohort (*n* = 14/100) vs. the OSU healthy controls (*n* = 29/402), *P* = 0.04 (Supplementary Table 1). We found that the frequency of this polymorphism in the control healthy population was 11% (43/402 cases) (38 heterozygous and 5 homozygous) (Supplementary Table 1 and Table 2). There were no differences with respect to the frequency of this polymorphism according to race or sex (Supplementary Table 2). The frequency of the miR-29 polymorphism was not significantly different between AML cases (*n* = 100: 16%) and controls (*n* = 402: 11%, *P* = 0.16). While not statistically significant, we found an enrichment for the presence of the miR-29 polymorphism in AML cases with CBF leukemia (*n* = 3/12: 25%) compared to controls (*n* = 43/402:11%) (Supplementary Table 1 and Figure 1C). To further explore this finding we screened for the presence of this polymorphism in a larger second cohort of 303 samples of newly diagnosed AML patients with t(8;21) (*n* = 131) and inv(16) (*n* = 172) obtained from the CALGB/Alliance leukemia tissue bank (See Tables 2 and 3 for patients characteristics). The polymorphism frequency was significantly higher in CBF leukemia (61/303, 20%) than healthy controls (43/402, 11%) of similar sex and race (*P <* 0.01) (Table 2 and Figure 1C). Only 8 of the 61 CBF-AML patients with polymorphism were homozygous for the finding. There were no significant differences in baseline clinical characteristics among inv(16) and t(8;21) patients with or without the polymorphism (Table 3), except in t(8;21) AML cases for age (median polymorphism 45 vs. median no polymorphism 37, *P* = 0.04), race (white polymorphism *n* = 17 (61%) vs. white no polymorphism *n* = 91 (90%) (*P <* 0.01); platelet count (median polymorphism 47 × 10^9^/L vs. median no polymorphism 36 × 10^9^/L, *P* = 0.03) and percentage of blasts in peripheral blood (median polymorphism 28 vs. median no polymorphism 39, *P* = 0.04).

**Table 1:** Patient characteristics for MD Anderson cohort

Characteristic	Value
**Age**	
Median	56
Range	17–82
**Sex, no.**	
Female	47
male	53
**Race, no**	
**White**	92
**Non-white**	8
Asian	1
Black	7
**Cytogenetics, no.**	
t(15;17)	1
inv(16)	10
t(8;21)	2
t(11q23)	4
Complex karyotype (CK)	11
isolated 7	5
Other cytogenetics	5
Normal Karyotype	62
*FLT3-ITD*	18
*NPM1*	34

**Table 2:** Patient characteristics and results for CALGB/Alliance cohort and controls

					^*^P values	^*^P values	^*^P values
	t(8;21)	inv(16)	All Cases	Controls	t(8;21) vs.	inv(16) vs.	All cases vs.
Characteristic	(*n* = 131)	(*n* = 172)	(*n* = 303)	(*n* = 402)	controls	controls	controls
**Age**							
Median	38	38	38	42	0.07	<0.01	<0.01
Range	(17–71)	(17–60)	(17–71)	(18–61)			
**Sex, no. (%)**							
Male	78 (60)	107 (62)	185 (61)	232 (58)	0.76	0.35	0.40
Female	53 (40)	65 (38)	118 (39)	170 (42)			
**Race, no. (%)**							
White	108 (84)	149 (89)	257 (87)	360 (91)	0.03^#^	0.53^#^	0.11^#^
Non-white	21 (16)	18 (11)	39 (13)	36 (9)			
Asian	4	0	4	11			
Black	13	16	29	25			
Other	2	2	4	0			
Unknown	2	5	7	6			
**Polymorphism, no (%)**							
Yes	28 (21)	33 (19)	61 (20)	43 (11)	<0.01	<0.01	<0.01
No	103 (79)	139 (81)	242 (80)	359 (89)			

^*^P-values for categorical variables were obtained using Fisher exact test, *P*-values for continuous variables are from Wilcoxon rank sum test.

^#^*P*-value is for White versus Non-white comparison.

**Table 3:** Clinical and molecular characteristics by polymorphism status in CALGB/Alliance t(8;21) and inv(16) AML patients

	t(8;21)			inv(16)		
	Polymorphism	No Polymorphism		Polymorphism	No Polymorphism	
Characteristic	(*n* = 28)	(*n* = 103)	^*^P values	(*n* = 33)	(*n* = 139)	^*^P values
**Age**						
Median	45	37	0.04	36	40	0.31
Range	(19–69)	(17–71)		(17–57)	(18–60)	
**Sex, no. (%)**						
Male	17 (61)	61 (59)	1.00	20 (61)	87 (63)	0.84
Female	11 (39)	42 (41)		13 (39)	52 (37)	
**Race, no. (%)**						
White	17 (61)	91 (90)	<0.01^#^	26 (81)	123 (91)	0.12^#^
Non-white	11 (39)	10 (10)		6 (19)	12 (9)	
Asian	3	1		0	0	
Black	6	7		6	10	
Other	0	2		0	2	
unknown	0	2		1	4	
**Hemoglobin (g/dl)**						
Median	8.7	8.8	0.92	9.4	8.7	0.07
Range	(5.9–13.3)	(3.5–12.7)		(6.3–12.3)	(3.1–14.8)	
**Platelet count (x10^9^/L)**						
Median	47	36	0.03	45	40	0.43
Range	(7–227)	(5–369)		(9–113)	(7–272)	
**WBC count (x10^9^)**						
Median	11.8	10.8	0.53	36.0	32.53	0.98
Range	(2.2–59.2)	(0.7–138.9)		(0.4–265)	(1.8–500)	
**% Blood blasts**						
Median	28	39	0.04	53	54	0.88
Range	(6–90)	(0–92)		(12–84)	(0–97)	
**% Bone Marrow blasts**						
Median	56	49	0.68	57	58	0.59
Range	(21–83)	(11–97)		(22–86)	(2–93)	
***C-KIT*, no. (%)**						
Mutated	8 (30)	24 (24)	0.62^#$^	9 (27)	34 (24)	0.82^$^
exon 17	5	17		2	11	
exon 8	2	6		6	21	
Both	1	1		1	2	
Wild-type	19 (70)	76 (76)		24 (73)	105 (76)	
unknown	1	3				

^*^P-values for categorical variables were obtained using Fisher exact test, *P*-values for continuous variables are from Wilcoxon rank sum test.

^#^*P*-value is for White versus Non-white comparison.

^$^*P*-value is for mutated versus wild-type comparison.

### Prognostic impact of the miR-29 polymorphism in CBF leukemia

To investigate whether this polymorphism has any prognostic impact in CBF-AML, we assessed the frequency of complete response (CR), event free-survival (EFS) and overall survival (OS) according to the miR-29 polymorphism status in a subset of *de novo* inv(16) (*n* = 126) and t(8;21) (*n* = 100) AML patients treated with intensive cytarabine/-anthracycline-based first-line therapy on CALGB/Alliance trials and whose clinical follow-up information was available.

First, we investigated whether the presence of the polymorphism predicts induction chemotherapy response. We did not observed any differences in the CR rates among the patients with and without the polymorphism; CR response rate for t(8;21) cases with miR-29 polymorphism *n* = 19 (90%) vs. cases with no miR-29 polymorphism *n* = 76 (96%) (*P* = 0.28); inv(16) cases with miR-29 polymorphism *n* = 25(100%) vs. no miR-29 polymorphism *n* = 98 (97%) (*P* = 1.00). Likewise, there were no statistical significant differences in EFS or OS between CBF patients with or without miR-29 polymorphism (Supplementary Figure 1A–1D). These results also held when considering patients with high risk CBF leukemia based on the presence of *c-KIT* mutation (Supplementary Figures 2A–2D and 3A–3D).

### The miR-29b-1/miR-29a cluster processing is affected by the T base deletion polymorphism

**Figure 2:**
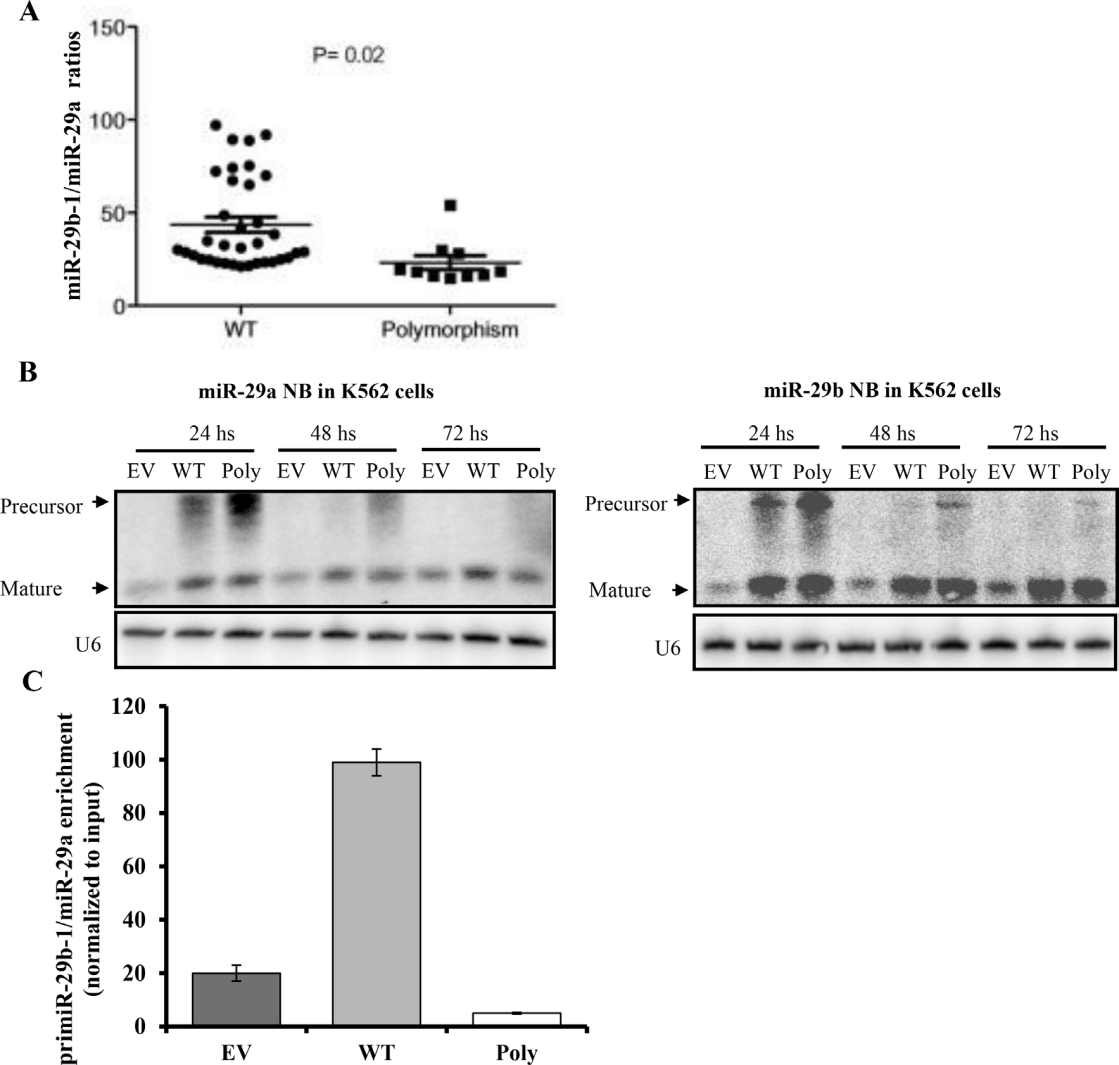
The miR-29b-1/miR-29a cluster processing is affected by the T base deletion polymorphism in acute myeloid leukemia (**A**) miR-29b-1/miR-29a expression ratios in wild type (WT) (*n* = 35) vs. polymorphism (Poly) cases (*n* = 10) as measured by quantitative real time RT-PCR. (**B**) Northern Blotting (NB) of miR-29b-1/miR-29a after transfection with empty vector (EV), WT or Poly constructs. U6 was used as loading control. (**C**) RNA immunoprecipitation assays in K562 cells transfected with miR-29b-1/miR-29a Poly, WT or EV construct.

To investigate whether this polymorphism affects miR-29b or miR-29a mature expression we measured miR-29b and miR-29a expression in 45 from the 100 primary AML samples (MD Anderson data set), where RNA was also available. Although mature miR-29a and miR-29b levels were not significantly different in polymorphism (*n* = 10) versus WT (*n* = 35) samples (data not shown), we observed that the miR-29a/miR-29b ratios were significantly lower in the polymorphism than WT samples (23.2 vs. 43.5 respectively, *P* = 0.02, *t*-test) (Figure 2A). These results suggest that the processing of this miRNA cluster is altered by the polymorphism. To confirm this possibility, we cloned the miR-29b-1/miR-29a cluster from 1 patient harboring the polymorphism into p-Retro Super plasmid and transfected into K562 cells (which have very low levels of endogenous miR-29 expression) along with WT (miR-29b-1/miR-29a cluster with no polymorphism) and empty vector constructs. Northern blotting performed 24 and 48 hours after transfection revealed an accumulation of the miR-29a precursor while the mature miR-29a level was decreased by 2 fold at 48 and 72 hours (Figure 2B and Supplementary Figure 4). While the precursor of miR-29b was also increased at 24 and 48 hours after transfection, the level of mature miR-29b was unchanged for all time points (up to 72 hours). Taken together, these data support that the polymorphism affects the processing of miR-29a. Given that Drosha complex is required for the processing of the primary transcript primiR-29b-1/miR-29a, we evaluated the efficiency of the interaction between the DROSHA complex and the primiR-29b-1/miR-29a by performing RIP assays in K562 ells transfected with either miR-29b-1/miR-29a polymorphism, miR-29b-1/miR-29a WT or empty vector construct. As shown in Figure 2C, the polymorphism showed reduced Drosha binding capacity when compared with the WT.

### The polymorphism dampens the targeting efficiency and tumor suppressor function of miR-29b-1/miR-29a cluster

**Figure 3:**
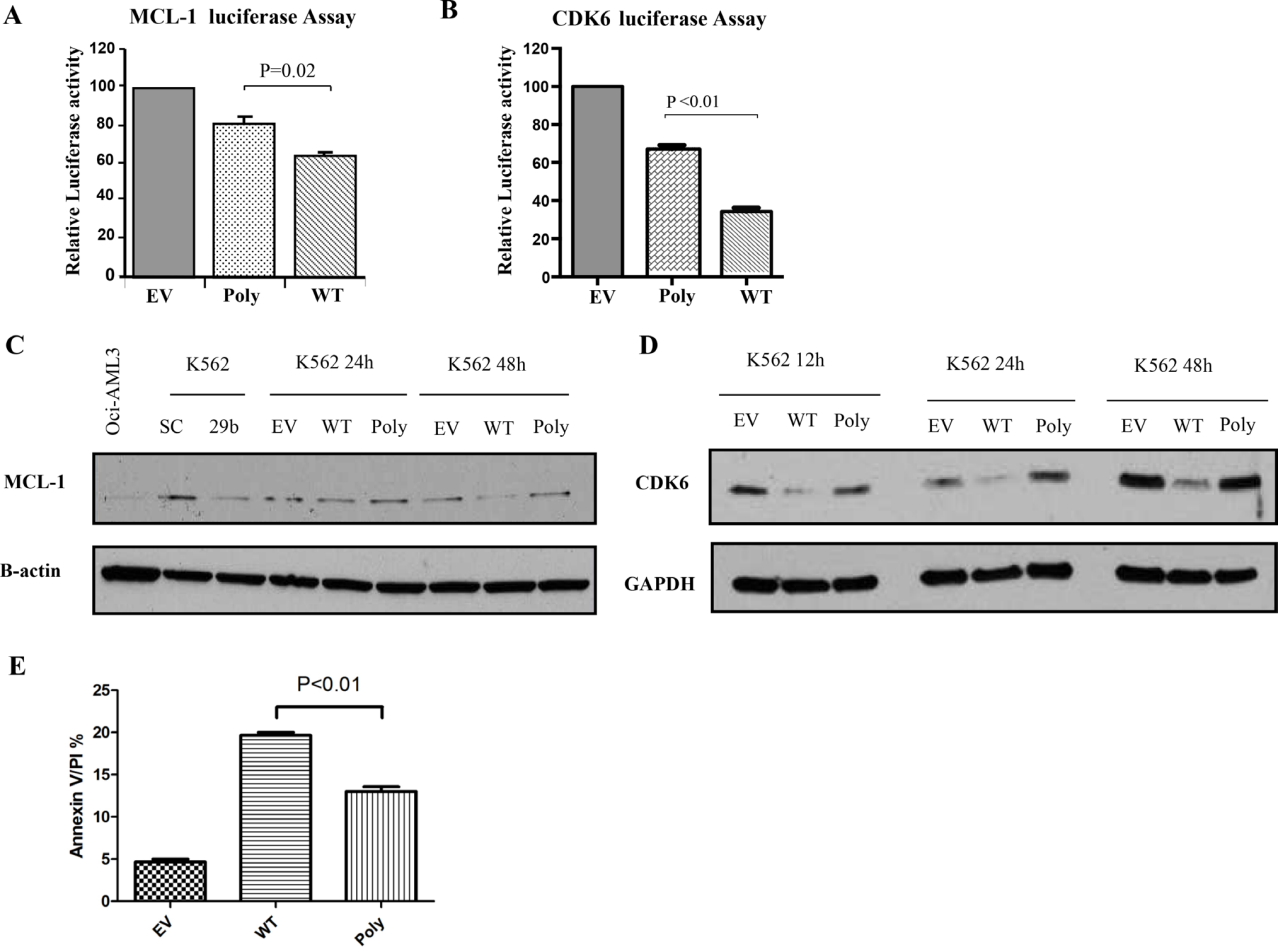
The polymorphism dampens the targeting efficiency and tumor suppressor function of the miR-29b-1/miR-29a cluster (**A**) MCL-1 and (**B**) CDK6 luciferase Assays. MiR-29b-1/miR-29a wild type (WT), polymorphism (Poly) or empty vector (EV) constructs were co-transfected with luciferase reporters containing the 3′untranslated regions (UTR) of MCL-1 or CDK6. The results are shown as relative luciferase values with respect to the EV after normalization with renilla. (**C**) Western Blotting for Mcl-1 and (**D**) Cdk6 proteins in K562 cells after transfection with miR-29b-1/miR-29a WT, Poly or EV constructs. GAPDH was used as loading control. As a control we also transfected cells with scramble (SC) or miR-29b (29b) oligonucleotides. (**E**) Apoptosis as measured using Annexin V/propidium iodine stain in K562 cells after transfection with miR-29b-1/miR-29a WT, Poly or EV constructs.

To assess whether this polymorphism affects miR-29 targeting efficiency, we co-transfected a reporter luciferase construct containing the 3′ untranslated region (3′UTR) of two known miR-29 target oncogenes, *MCL-1* and *CDK6* [21] with the WT, empty vector and polymorphism harboring miR-29b-1/miR-29a cluster and performed luciferase assays. Interestingly, relative normalized luciferase activities were less inhibited with the polymorphism cluster than the WT construct for *MCL-1* (relative reduction WT: 63%, polymorphism: 80%, *P* = 0.02, *t*-test) (Figure 3A) and for *CDK6* (relative reduction WT: 37%, polymorphism: 68%, *P <* 0.01, *t*-test) (Figure 3B). Next, we investigated whether the effects observed at the luciferase assays were translated to the protein level. Indeed, we showed that Mcl-1 and Cdk6 protein down-regulation elicited by the ectopic WT cluster overexpression was stronger than the one observed for the cluster harboring the polymorphism (Figure 3C and 3D). Finally, we investigated whether the less efficient reduction in Mcl-1 protein expression caused by the polymorphism was functionally important. Since Mcl-1 is an anti-apoptotic protein [21], we compared apoptosis induction between the WT and polymorphism miR-29b-1/miR-29a cluster over-expression in K562 cell lines. In Figure 3E, we showed that the miR-29b-1/miR-29a cluster harboring the polymorphism exhibited less apoptosis that the WT with respect to controls (empty vectors).

## DISCUSSION

Our results identify a germline polymorphism within the miR-29b-1/miR-29-a cluster, whose frequency is increased in the subset of CBF-AML patients with respect to the normal population. These results were further validated using a second independent large cohort of 303 de novo CBF-AML patients (including both inv(16) and t(8;21)). The causes for such an increased frequency of this polymorphism in this AML subset are unknown. Interestingly, miR-29b plays a critical role in *c-KIT* oncogene expression regulation in CBF leukemia by dampening *c-KIT* oncogene transcription through the direct targeting of its activator Sp1 [20]. Ectopic overexpression of miR-29b in CBF-cell lines and in a murine AML model represses *c-KIT* expression and induced cell death [20]. The fact that the miR-29 polymorphism is more prevalent in CBF leukemia, further support the important functional role of miR-29 in CBF-leukemia.

Another interesting finding from our work was the finding of this polymorphism in patients with isolated chromosome 7 monosomy (1/6). We have previously shown that patients with monosomy 7 and del7q primary AML blasts, have lower miR-29a and miR-29b expression levels than AML blasts without any chromosome 7 abnormalities, indicating that the structural genomic deletion is responsible for the decreased miRNA expression since this cluster is located in chromosome 7q32 [21]. The finding of a polymorphism that affects the expression and function of this cluster in the other allele of AML patients with monosomy 7 is intriguing and may contribute to further inactive the tumor suppressor functions of the miR-29 cluster.

Mechanistically, this polymorphism affects the expression ratio of mature miR-29b and miR-29a by dampening the processing of miR-29a. Northern blotting showed accumulation of miR-29 precursors with an associated decrease in the expression of mature miR-29a. The levels of mature miR-29b however, were unchanged with respect to the WT controls. Interestingly, the polymorphism is between miR-29b-1 and miR-29a and after the miR-29b transcription initiation site, a possible explanation as to why the polymorphism does not have an effect on mature miR-29b. In the presence of reduced mature miR-29a, the accumulation of the miR-29a precursor suggests that a problem exists in the processing of miRNA. Previous groups including our own have shown that mutations or polymorphisms affecting the precursor miRNA can affect the miRNA processing and results in precursor accumulation and decreased mature miRNA expression [14, 28]. Notably, in the case of the polymorphism described here the deletion is outside the miRNA precursor, indicating that the genomic region between the two miRNAs is important for miRNA processing of one of the mature miRNAs in the cluster.

The aberrant ratios between miR-29a and miR-29b resulting from the polymorphism found in the experimental studies were also confirmed in primary AML samples. This finding raises the question about the significance and contribution of the different expression levels among members of this cluster to the functions of miR-29 in normal physiological conditions and in disease. We previously found significant differences in the expression of genes correlated with miR-29a or miR-29b expression in primary AML samples, indicating non-overlapping functions for miR-29a and miR-29b in AML [21]. It is likely that a tight balance between the expression of both mature miR-29a and miR-29b exists and alterations in the expression ratios between them may affect the functions of both miRNAs. Supporting this, we showed that this polymorphism negatively impacts the ability of this cluster to target the oncogenes *MCL-1* and *CDK6*, both known miR-29 targets.

In summary, we discovered a polymorphism in the miR-29b-1/miR-29a cluster that is enriched in CBF-AML cases. This polymorphism affects miR-29b-1/miR-29a cluster processing, resulting in accumulation of the precursor, and low levels of mature miR-29a. Functionally, the polymorphism impacts the ability of the cluster to target *MCL-1* and *CDK6* and induce apoptosis.

## MATERIALS AND METHODS

### Patients samples

Frozen diagnostic BM or (PB) samples were obtained from adult AML patients from the MD Anderson Tissue bank (*n* = 100) and from the Cancer and leukemia group B (CALGB)/Alliance leukemia tissue bank (*n* = 303). Cytogenetic analyses of the samples were performed at diagnosis, using unstimulated short-term (24-, 48-, and 72-hour) cultures with or without a direct method and G-banding. For the CALGB/-Alliance dataset, cytogenetic analyses at diagnosis were performed by CALGB–approved institutional cytogenetic laboratories as part of the cytogenetic companion study 8461 and confirmed by central karyotype review. The criteria used to describe a cytogenetic clone and description of karyotype followed the recommendations of the International System for Human Cytogenetic Nomenclature [23]. *FLT3-ITD*, *FLT3* activation loop *D835* and *NPM1* mutations analyses were performed on most of the samples using Sanger sequencing as previously described [8]. All patients gave informed consent for cryopreservation and use of the samples for molecular studies. Approval was obtained from the institutional board review from the MD Anderson Cancer Center and The Ohio State University. DNA from the blood of 402 control healthy subjects was obtained from the Ohio State University Human Genetics Sample Bank.

### Mutation screening

To screen for mutations or polymorphisms, the entire genomic region from primary AML samples, corresponding to the miR-29b-1/miR-29-a cluster precursor, located in chromosome 7q32, including 200 bp at the 5′ and 3′ ends was amplified and sequenced using the Applied Biosystems DNA sequencing system (Applied Biosystems, Foster City, CA). When a deviation from the normal sequence was found, a panel of DNA from the blood of 402 control subjects was screened to identify polymorphisms.

### Real-Time quantification of miRs and target genes

The single tube TaqMan miRNA assays were used to detect and quantify mature miR-29a and miR-29b expression as previously described [24] using PCR 9700 Thermocycler ABI Prism 7900HT and the sequence detection system (Applied Biosystems). To detect the Myeloid Cell Leukemia sequence 1 (*MCL-1*) and the Cyclin-Dependent Kinase 6 (*CDK6*) mRNA levels, we used the TaqMan gene expression assays. Normalization was performed with 18s and U6. Comparative real-time PCR was performed in triplicate, including no-template controls. Relative expression was calculated using the comparative Ct method [25].

### Cell transfection with miRNA expression vectors and miRNA precursors

The genomic region corresponding to the miR-29b-1/miR-29a cluster (200 bp before and after precursor) from a wild type (WT) or a polymorphism patient was cloned into the pSuperRetro expression vector (OligoEngine, Seattle, WA). The miRNA precursor miR-29b was purchased from Ambion (Austin, TX). Five million K562 cells (ATCC, Manassas, VA) were nucleoporated using AMAXA (Gaithersburg, MD) with 5ug of vectors pSuperRetro-miR-29a-1/miR-29b-WT, pSurperRetro-miR-29a-1/miR-29b-Polymorphism and empty vector or with 5 ug of precursor oligonucleotides (miR-29b or scrambled controls) in a total volume of 10 ml.

### Northern blotting

Northern Blotting was performed as previously described [19]. Briefly, total RNA was extracted using Trizol reagent (Invitrogen, Carlsbad, CA). RNA samples (10 ug) were run on 12% polycramide denaturing (urea) precast gels (BIO-RAD, Hercules, CA) and then transfered onto Hybond membrane (Amersham Pharmacia Biotech, Uppsala, Sweden). The hybridization was performed with α-32P miR-29a and -b labeled probes overnight at 42°C. The miR-29 probes sequences were the complementary one to the mature miR-29s. As a loading control we measured U6 expression after stripping the filter as previously described [19].

### RNA immunoprecipitation assay (RIP)

RIP was performed with the Magna RIP kit (Millipore, Billerica, MA) according to the manufacturer's instruction. Cells transfected with vectors pSuperRetro-miR-29a-1/miR-29b-WT, pSurperRetro-miR-29a-1/miR-29b-Polymorphism and empty vector, were lysed and the RNA associated proteins were immunoprecipitated (IP) with Drosha Antibody Cat. Number A301-886A (Bethyl Laboratories, Montgomery, TX). RNA was extracted from the Drosha immunoprecipitated complex by following the protocol provided by the kit and quantified with Nanodrop2000 (Thermo-Scientific, Waltham, MA). Total RNA (25 ng) was retro-transcribed with High Capacity cDNA Reverse Transcription Kit (Lifetech, Carlsbad, CA). The primiR-29b-1/miR-29a exogenous expression was assessed by quantitative real time PCR (SYBR green, Qiagen, Hilden, Germany) and referenced to the non-immunoprecipitated control (Input) using the following primers: primiR-29a1/miR-29b Fwd: 5′- CAT ATA TCA CAA ATG GCA GTC AGG TCT -3′ and Rev: 5′- TGT ACA GGA TAT CGC ATT GTT GGA A-3′. Real time PCRs were performed in an Applied Biosystems 7500 instrument. MiRNA expression was measured using Ct (threshold cycle).

### Statistical analysis

Fisher's exact test and *t*-test were used to compare baseline characteristics and average miRNA expression between groups of patients [26]. These reported *P* values were two-sided and obtained using the SPSS software package (SPSS 10.0). Baseline demographic, clinical, and molecular features were compared between the Alliance/CALGB cohort and controls and between polymorphism and no polymorphism using the Wilcoxon rank sum and Fisher's exact tests for continuous and categorical variables, respectively [26]. The estimated probabilities of event-free (EFS) and overall survival (OS) were calculated using the Kaplan–Meier method, and the log-rank test evaluated differences between survival distributions [27]. Clinical endpoint definitions are given in the Data Supplement. All statistical analyses were performed by the Alliance Statistics and Data Center on a database locked on June 16, 2016 using SAS 9.4 and TIBCO Spotfire S+ 8.2.

### Luciferase reporter experiments

The 3′ UTR segment containing the target sites for miR-29b from the *MCL-1* gene was amplified by PCR from genomic DNA and inserted into the pGL3 control vector (Promega, Madison, WI), using the XBA1 site immediately downstream from the stop codon of luciferase. The following primer sets were used to generate specific fragments: *MCL-1* FW 5′TGGAACTCATT AGCTGTGTGC3′, and RW 5′GATGCCAATGCAAAA ACTTG 3′. We also generated an insert with deletions of 4 bp from the site of perfect complementarity using the Quiagen XL-site directed Mutagenesis Kit. Wild type and mutant insert were confirmed by sequencing. Human cell line K562 was grown in 10% fetal bovine serum in RPMI-1640. The cells were cotransfected in 10 ml plates using nucleoporation (Amaxa) according to the manufacturer's protocol using 5 ug of the firefly luciferase report vector and 0.5 ug of the control vector containing Renilla luciferase, pRL–TK (Promega) along with 5 ug of the pSuperRetro-miR-29a-1/miR-29b-WT, pSurperRetro-miR-29a-1/miR-29b-Polymorphism or empty vector. Firefly and Renilla luciferase activities were measured consecutively using the dual luciferase assays (Promega) 24 hrs after transfection.

### Apoptosis experiments

Annexin V/Propidium iodide stain (BD Pharmingen, San Diego, CA) was performed at different time points after nucleoporation with pSuperRetro expression vectors as described above.

### Western blotting

Total protein extracts from K562 transfected cells with pSuperRetro expression vectors were extracted using RIPA buffer (SIGMA, St Louis, MO). Protein expression was analyzed by Western blotting using Mcl-1, Cdk6 and GAPDH (Santa Cruz, Santa Cruz, CA).

## SUPPLEMENTARY MATERIALS FIGURES AND TABLES


