# Supplementary material for: Discovery and functional implications of a miR-29b-1/miR-29a cluster polymorphism in acute myeloid leukemia
Source: Oncotarget. 2017 Dec 12;9(4):4354–65. doi: 10.18632/oncotarget.23150 (PMC5796978; doi:10.18632/oncotarget.23150)
Supplement: Supplementary file 1 [file oncotarget-09-4354-s001.pdf]

Discovery and functional implications of a miR-29b-1/miR-29a cluster polymorphism in acute myeloid leukemia

SUPPLEMENTARY MATERIALS

Participating institutions

The following Cancer and Leukemia Group B (CALGB)/Alliance for Clinical Trials in Oncology (Alliance) institutions participated in this study and contributed at least two patients. For each of these institutions, the current or last principal investigators are listed as follows: The Ohio State University Medical Center, Columbus, OH: Richard M. Goldberg; Wake Forest University School of Medicine, Winston-Salem, NC: Heidi Klepin; Washington University School of Medicine, St. Louis, MO: Nancy L. Bartlett; Dana Farber Cancer Institute, Boston, MA: Harold J. Burstein; North Shore University Hospital, Manhasset, NY: Daniel R. Budman; Roswell Park Cancer Institute, Buffalo, NY: Ellis G. Levine; University of Chicago Medical Center, Chicago, IL: Hedy L. Kindler; University of Iowa Hospitals, Iowa City, IA: Daniel A. Vaena; University of North Carolina, Chapel Hill, NC: Thomas C. Shea; Ft. Wayne Medical Oncology/Hematology, Ft. Wayne, IN: Sreenivasa Nattam; University of Maryland Cancer Center, Baltimore, MD: Martin J. Edelman; Christiana Care Health Services, Inc., Newark, DE: Gregory Masters; Dartmouth Medical School, Lebanon, NH: Konstantin Dragnev; Duke University Medical Center, Durham, NC: Jeffrey Crawford; University of Vermont Cancer Center, Burlington, VT: Claire Verschraegen; Eastern Maine Medical Center, Bangor, ME: Thomas H. Openshaw; Mount Sinai School of Medicine, New York, NY: Lewis R. Silverman; Weill Medical College of Cornell University, New York, NY: Scott Tagawa; University of Massachusetts Medical Center, Worcester, MA: William V. Walsh; Western Pennsylvania Hospital, Pittsburgh, PA: John Lister; University of Puerto Rico School of Medicine, San Juan, PR: Eileen I. Pacheco; SUNY Upstate Medical University, Syracuse, NY: Stephen L. Graziano; University of Alabama at Birmingham: Robert Diasio; Rhode Island Hospital, Providence, RI: Howard Safran; University of Illinois, Chicago, IL: Arkadiusz Z. Dudek; Moores University of California San Diego Cancer Center, San Diego, CA: Barbara A. Parker; Walter Reed National Military Medical Center, Bethesda, MD: Mary Kwok; Virginia Commonwealth University, Richmond, VA: Steven Grossman; University of Missouri/Ellis Fischel Cancer Center, Columbia, MO: Clint Kingsley; University of Tennessee Cancer Center, Memphis, TN: Harvey B. Niell; University of Nebraska Medical Center, Omaha, NE: Apar Ganti.

Treatment protocols

All patients included in our study were treated on (CALGB)/Alliance first-line protocols for patients with acute myeloid leukemia (AML), and received cytarabine/daunorubicin-based induction therapy. Per protocol, all patients were to receive at least one induction cycle. For patients with residual leukemia present in a bone marrow (BM) biopsy after one induction cycle, a second cycle of induction was administered. None of the protocols included allogeneic stem cell transplantation in first complete remission (CR). Patients enrolled on the treatment protocols also provided written informed consent to participate in the companion protocols CALGB 20202 (molecular studies in AML), CALGB 8461 (prospective cytogenetic companion), and CALGB 9665 (leukemia tissue bank), which involved collection of pretreatment BM aspirates and blood samples.

Of the 303 enrolled patients, 77 patients were excluded in outcome analyses due to early induction deaths or did not receive cytarabine as consolidation therapy. Two hundred and twenty [100 with t(8;21) and 126 with inv(16)] received treatment and were included in outcome analyses. Details of the therapeutic schemas for the CALGB protocols have been reported previously [1–4]. The majority of the patients received one of the following induction regimens: 1) cytarabine 100 mg/m^2/d × 7 days in combination with daunorubicin and etoposide × 3 days ± PSC-833, a multidrug resistance modulator (ADE ± P) [128 patients on CALGB 9621 and 19808] or 2) cytarabine 200 mg/m^2/d × 7 days in combination with daunorubicin 45 mg/m^2/d × 3 days (AD) [27 patients on CALGB 8525 and 9222] or 3) daunorubicin 90 mg/m^2 for 3 days, etoposide 100 mg/m^2 for 3 days, and cytarabine 100 mg/m^2 for 7 days (3 + 3 + 7) [60 patients on CALGB 10503] or 4) cytarabine 100 mg/m^2/d × 7 days in combination with daunorubicin 60 mg/m^2/d × 3 days and +/- dasatinib 100 mg/d on days 8–21 [9 patients on CALGB 10801] or midostaurin 50 Mg days 8–22 [2 patients on CALGB 10603].

As part of consolidation, all patients received cytarabine, according to one of the following doses and schedules: (a) 100 mg/m^2/d for 5 days × 4 courses; (b) 400

mg/m^2/d for 5 days × 4 courses and (c) 3 g/m^2 every 12 hours on days 1, 3, and 5 × 3 or 4 courses.

Definition of clinical endpoints

Clinical endpoints were defined according to generally accepted criteria [5]. CR required a BM aspirate with cellularity >20% with maturation of all cell lines, <5% blasts and undetectable Auer rods; in peripheral blood, an absolute neutrophil count of ≥1.5 × 10^9/L, platelet count of >100 × 10^9/L, and leukemic blasts absent; and no evidence of extramedullary leukemia, all of which had to persist for ≥4 weeks [5]. Relapse was defined by the presence of ≥5% BM blasts, or circulating leukemic blasts, or the development of extramedullary leukemia. Overall survival (OS) was measured from the date of study entry until the date of death (from any cause); patients alive at last follow-up were censored. Event-free survival (EFS) was measured from the date of study entry until the date of failure to achieve CR, relapse or death. Patients alive and in CR at last follow-up were censored.

REFERENCES

1. Kolitz JE, George SL, Dodge RK, Hurd DD, Powell BL, Allen SL, Velez-Garcia E, Moore JO, Shea TC, Hoke E, Caligiuri MA, Vardiman JW, Bloomfield CD, Larson RA, and Cancer and Leukemia Group B. Dose escalation studies of cytarabine, daunorubicin, and etoposide with and without multidrug resistance modulation with PSC-833 in untreated adults with acute myeloid leukemia younger than 60 years: final induction results of Cancer and Leukemia Group B Study 9621. J Clin Oncol. 2004; 22:4290–301. https://doi.org/10.1200/JCO.2004.11.106.
2. Kolitz JE, George SL, Marcucci G, Vij R, Powell BL, Allen SL, DeAngelo DJ, Shea TC, Stock W, Baer MR, Hars V, Maharry K, Hoke E, et al, and Cancer and Leukemia Group B. P-glycoprotein inhibition using valspodar (PSC-833) does not improve outcomes for patients younger than age 60 years with newly diagnosed acute myeloid leukemia: Cancer and Leukemia Group B study 19808. Blood. 2010; 116:1413–21. https://doi.org/10.1182/blood-2009-07-229492.
3. Blum W, Donohue K, Marcucci G, DeAngelo DJ, Uy GL, Powell BL, Stock W, Baer M, Kolitz JE, Wetzler M, Hoke E, Moser BK, Larson RA. Challenges for Conducting Clinical Trials Evaluating Maintenance Chemotherapy In Acute Myeloid Leukemia (AML): a Cancer and Leukemia Group B (CALGB) Study. Blood. 2010; 116:2176.
4. Marcucci G, Geyer S, Zhao J, Caroll AJ, Bucci D, Vij R, Blum W, Pardee T, Wetzler M, Stock W, Bloomfield CD, Larson RA, Stone RM. Adding The KIT Inhibitor Dasatinib (DAS) To Standard Induction and Consolidation Therapy For Newly Diagnosed Patients (pts) With Core Binding Factor (CBF) Acute Myeloid Leukemia (AML): Initial Results Of The CALGB 10801 (Alliance) Study. Blood. 2013; 122:357.
5. Cheson BD, Cassileth PA, Head DR, Schiffer CA, Bennett JM, Bloomfield CD, Brunning R, Gale RP, Grever MR, Keating MJ. Report of the National Cancer Institute-sponsored workshop on definitions of diagnosis and response in acute myeloid leukemia. J Clin Oncol. 1990; 8:813–9. https://doi.org/10.1200/JCO.1990.8.5.813.

Supplementary Table 1: Patient characteristics for MD Anderson AML cohort and controls

Characteristic	AML cases (*n* = 100)	Controls (*n* = 402)	*P*
**Age**			
Median	56	42	<0.01
Range	(17–82)	(18–61)	
**Sex, no. (%)**			
Female	47 (47)	170 (42)	0.43
male	53 (53)	232 (58)	
**Race, no. (%)**			
White	92 (92)	360 (91)	0.85
Non-white	8 (8)	36 (9)	
Asian	1	11	
Black	7	25	
Other	0	0	
Unknown	0	6	
**Polymorphism, no. (%)**			
yes	16 (16)	43 (11)	0.16
no	84 (84)	359 (89)	
**CBF polymorphism, no. (%)**			
yes	3 (25)	43 (11)	0.14
no	9 (75)	359 (89)	

CBF: core binding factor

Supplementary Table 2: Patient characteristics comparison for controls (*n* = 402)

Characteristic	Polymorphism (*n* = 43)	No Polymorphism (*n* = 359)	**P*-value
**Age**			0.65
Median	42	42	
Range	(18–59)	(18–61)	
**Sex, no. (%)**			0.75
Female	17 (40)	153 (43)	
Male	26 (60)	206 (57)	
**Race, no (%)**			1.00#
White	39 (93)	321 (91)	
Non-white	3 (7)	33 (9)	
Asian	0	11	
Black	3	22	
Unknown	1	5	

**P*-values for categorical variables were obtained using Fisher exact test, *P*-values for continuous variables are from Wilcoxon rank sum test.
#*P*-value is for White versus Non-white comparison.

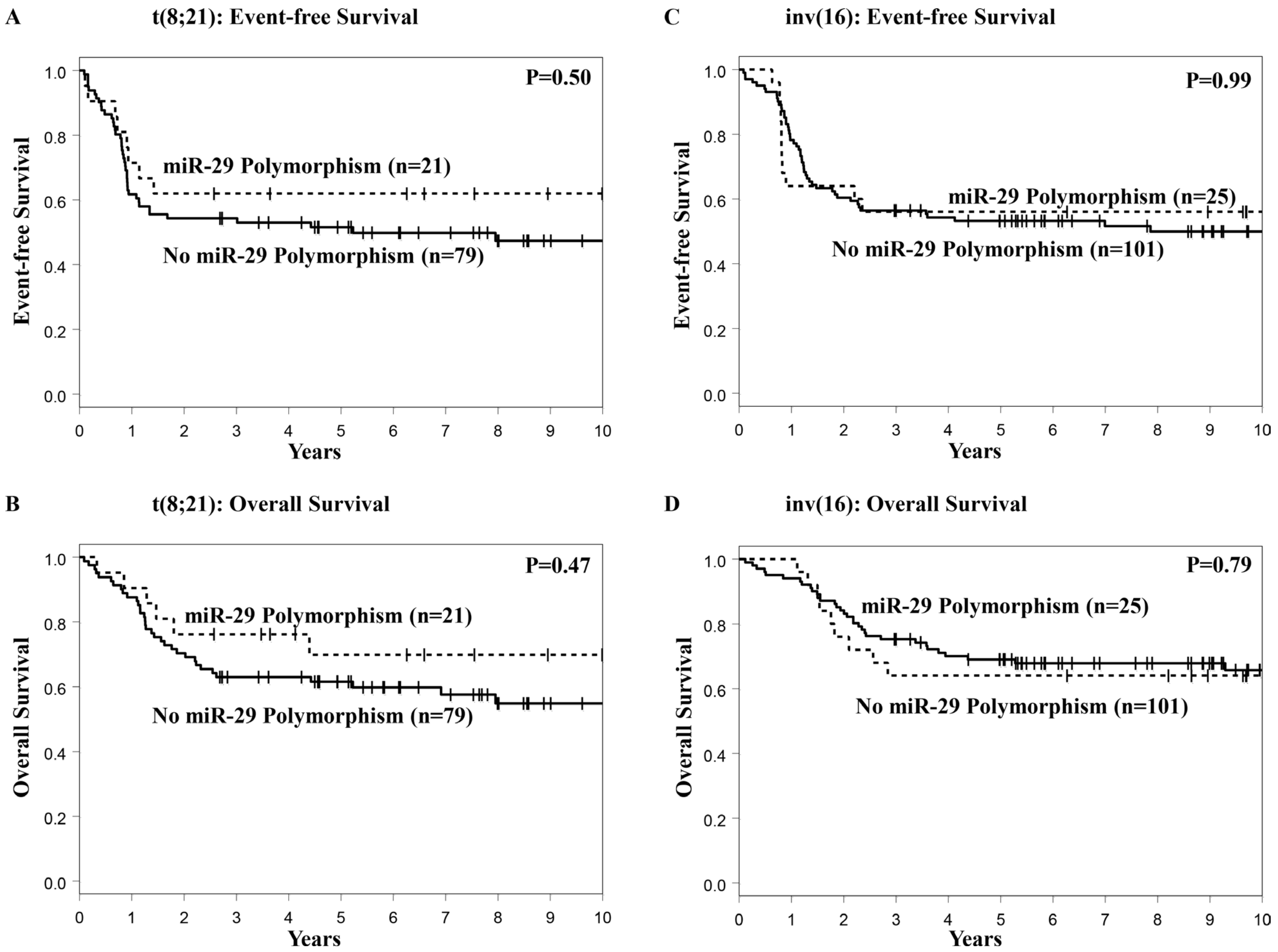

Supplementary Figure 1: Outcome of acute myeloid leukemia patients with de novo t(8;21) and inv(16) according to the presence of the miR-29 polymorphism. (**A**) Event-free survival and (**B**) Overall Survival in t(8;21) patients. (**C**) Event-free survival and (**D**) Overall Survival in inv(16) patients.

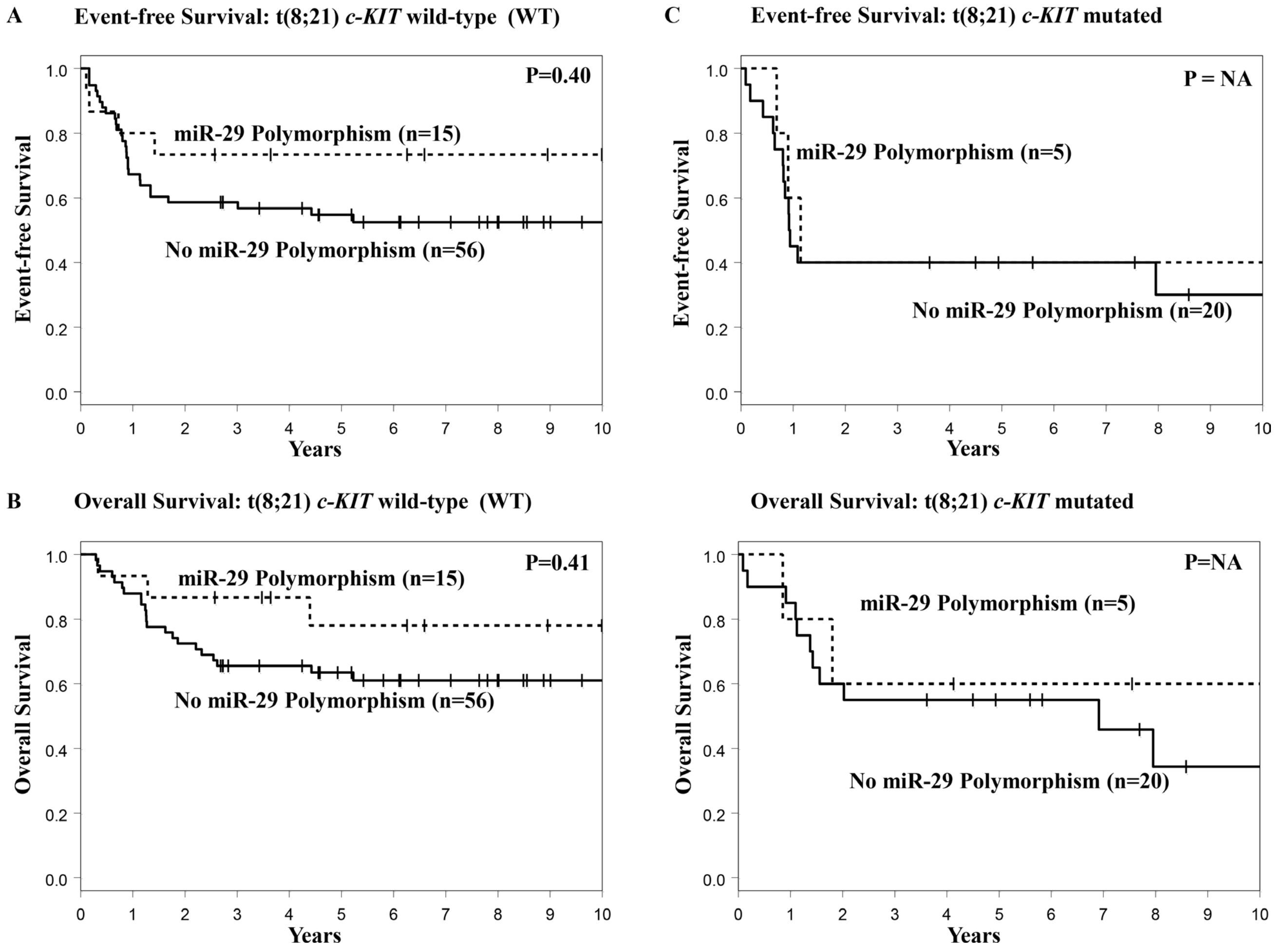

Supplementary Figure 2: Outcome of acute myeloid leukemia patients with de novo t(8;21) according to the presence of the miR-29 polymorphism and *c-KIT* mutation status. (**A**) Event-free survival and (**B**) Overall Survival in t(8;21) patients with wild type *c-KIT*. (**C**) Event-free survival and (**D**) Overall Survival in t(8;21) patients with mutated *c-KIT*. NA means P-value cannot be calculated because sample size is too small.

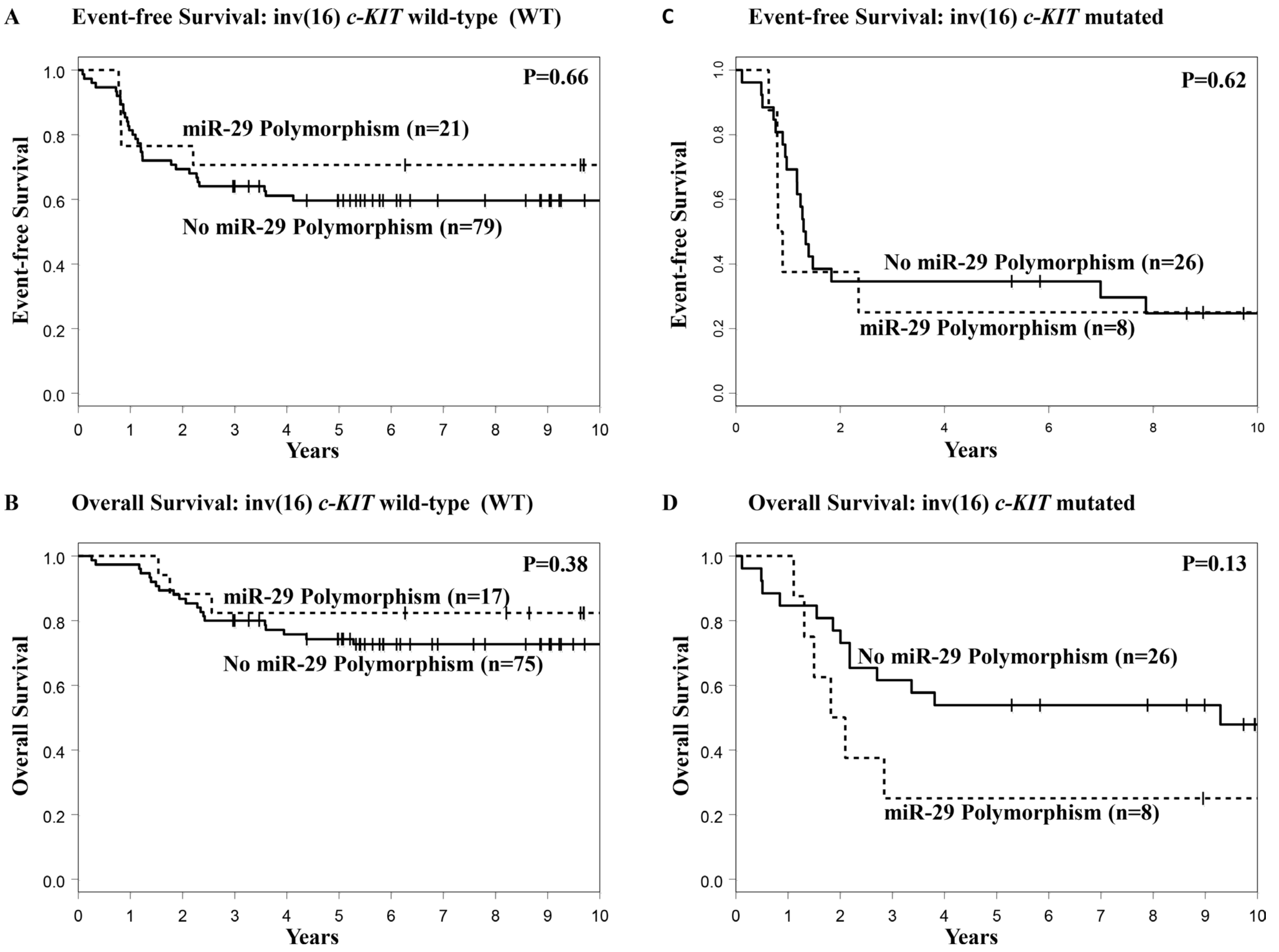

Supplementary Figure 3: Outcome of acute myeloid leukemia patients with de novo inv(16) according to the presence of the miR-29 polymorphism and *c-KIT* mutation status. (**A**) Event-free survival and (**B**) Overall Survival in inv(16) patients with wild type *c-KIT*. (**C**) Event-free survival and (**D**) Overall Survival in inv(16) patients with mutated *c-KIT*.

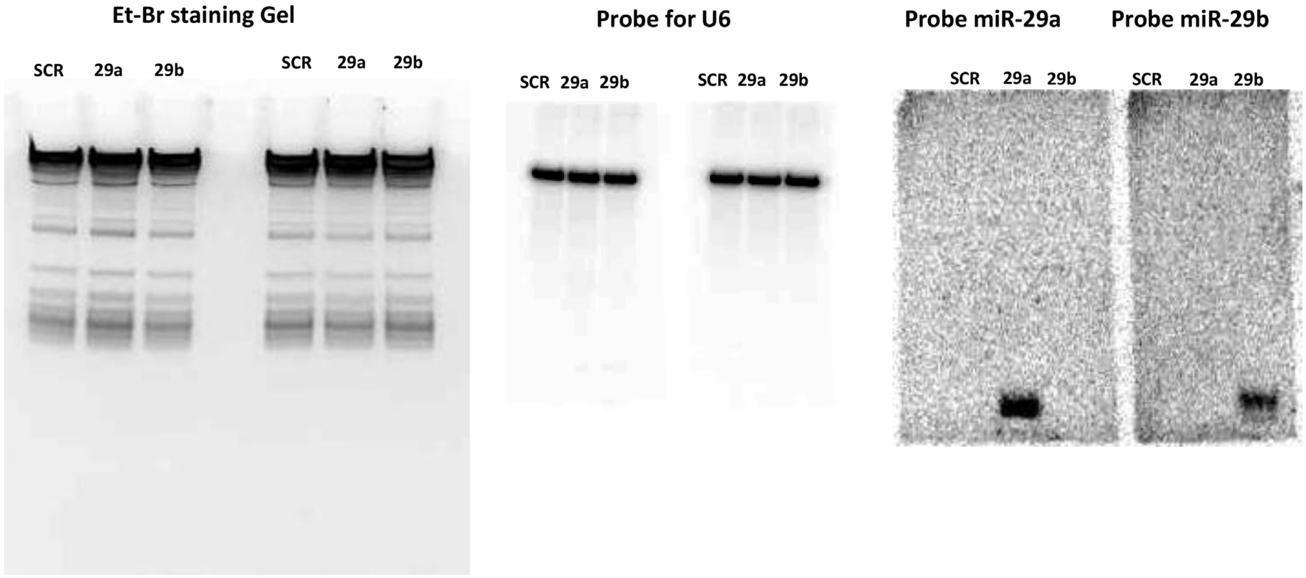

Supplementary Figure 4: Northern Blots miR-29 probe specificity. K562 Cells Transfected with miR-29a, miR-29b, and scramble to confirm the specificity of the Northern blot probes.
